# Lamotrigine Induced DRESS Syndrome in a Child: A Case Report and Literature Review

**DOI:** 10.3390/children8111063

**Published:** 2021-11-19

**Authors:** Chien-Heng Lin, Sheng-Shing Lin, Syuan-Yu Hong, Chieh-Ho Chen, I-Ching Chou

**Affiliations:** 1 Division of Pediatric Pulmonology, China Medical University Children’s Hospital, Taichung 404327, Taiwan; lch227@ms39.hinet.net (C.-H.L.); d30270@mail.cmuh.org.tw (C.-H.C.); 2 Department of Biomedical Imaging and Radiological Science, College of Medicine, China Medical University, Taichung 404328, Taiwan; 3 Division of Pediatric Neurology, China Medical University Children’s Hospital, Taichung 404327, Taiwan; d3900@mail.cmuh.org.tw (S.-S.L.); dazingdog@hotmail.com (S.-Y.H.); 4 Graduate Institute of Integrated Medicine, China Medical University, Taichung 404328, Taiwan

**Keywords:** DRESS syndrome, lamotrigine, dyspnea

## Abstract

Lamotrigine is an important anticonvulsant drug. Its use, however, has been limited by the risk of potentially life-threatening dermatological reactions, such as a drug reaction with eosinophilia and systemic symptoms (DRESS). Here, we report the case of a 7-year-6-month-old girl with a history of epilepsy who developed a skin rash with dyspnoea after 2 weeks of lamotrigine treatment, with DRESS ultimately being diagnosed. After discontinuation of the offending drug and the initiation of systemic glucocorticosteroids, the DRESS symptoms were relieved and the patient was discharged in a stable condition. Anticonvulsant drugs such as lamotrigine are among the factors that induce DRESS in children. When a patient displays skin rash and systemic organ involvement following the initiation of an anticonvulsant drug, DRESS should not be overlooked as a diagnosis, and immunosuppressant drugs should be considered as an option for treating DRESS patients.

## 1. Introduction

Anticonvulsant drugs can cause adverse cutaneous reactions, such as drug-induced hypersensitivity syndrome (DIHS), which is characterized by a skin rash, fever, and the involvement of internal organs, mainly the liver, kidneys, and lungs. The symptoms of DIHS usually develop 2–6 weeks after starting the offending drug but may occur at any time.

Bocquet et al. extended the definition of DIHS and introduced the term DRESS (drug reaction with eosinophilia and systemic symptoms) [1]. The drugs most commonly responsible for inducing DRESS include anticonvulsant drugs (carbamazepine, phenobarbital, phenytoin, and lamotrigine), antibiotics (minocycline, ß-lactams, and sulfonamides), antiviral agents, dapsone, sulfasalazine, and allopurinol [2,3]. The incidence of DRESS has been estimated to be between 1/1000 and 1/10,000 exposures to anticonvulsant drugs [4]. Furthermore, DRESS has previously been reported to be associated with herpesviruses, although existing explanations of how viral infections contribute to the pathogenesis of DRESS remain speculative [5].

There are two sets of diagnostic criteria for the diagnosis of DRESS; one set consists of the RegiSCAR criteria and the other consists of the SCAR-J criteria developed by Japanese investigators [6,7,8]. The treatment for DRESS consists of immediate withdrawal of the culprit drug followed by the initiation of systemic steroids.

## 2. Case Presentation

**Figure 1:**
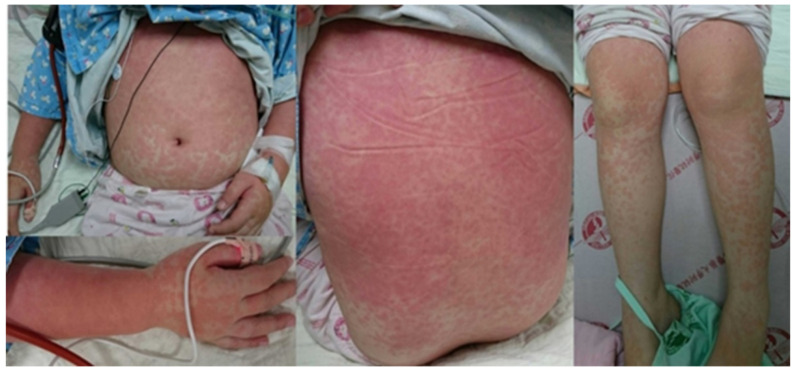
Scattered red maculopapular rash on the trunk and limbs, partially pressed to fade and partially fused into patches.

A 7-year-6-month-old girl was brought to our emergency room (ER) after experiencing a fever and dyspnea for 3 days. In the ER, a physical examination revealed lymphadenopathy, mild injected throat with coarse breathing sound, and a maculopapular skin rash on her face, trunk, and limbs (Figure 1). There were no specific findings upon neurological examination.

Lab data for the patient showed leukocytosis (white blood cell count: 18,100/uL) with an elevated level of eosinophils (10%, 1810/uL), with 49.4% neutrophils, 29.2% lymphocytes, and 11% monocytes. The patient’s C-reactive protein level was 5.3 mg/dL (normal: <0.8 mg/dL), while her aspartate transaminase (AST) level and alanine aminotransferase (ALT) level were 253 U/L and 93 U/L, respectively. A mycoplasma rapid test was positive, and a chest X-ray showed bilateral perihilar lung bronchitis infiltrates. Therefore, bronchopneumonia was suspected initially. After admission, the patient suffered from progressive dyspnea, and then was transferred to the pediatric intensive care unit for non-invasive ventilator support with bi-level positive airway pressure support. The laboratory data obtained after admission revealed that EB VCA and EBVA IgG were both positive, but the adenovirus rapid tests, HSV I/II IgM, EB VCA IgM, and mycoplasma IgM were all negative, and the mycoplasma pneumonia IgG test was equivocal.

In tracing back her past history, it was discovered that she had a history of epilepsy, which was kept under control with an anticonvulsant drug (Depakine, at an initial dose of 250 mg qhs). However, her electroencephalography (EEG) results showed generalized epileptiform discharge, and the dose of Depakine was increased to q12h one year ago, at which time her seizures went into remission. However, her body weight changed from 35 kg to 42.5 kg, resulting in her being overweight (body mass index > 24). Therefore, the anticonvulsant drug was changed to lamotrigine 25 mg and then 50 mg q12h one month prior to her arrival at the ER. The patient reported experiencing a maculopapular skin rash accompanied by an itchy sensation one week prior to her arrival in the ER, which was untreated because it was not obvious at the time.

Because of her clinical presentation of an erythematous rash spread all over her body about 2 weeks after starting lamotrigine, lamotrigine-induced drug reaction with eosinophilia and systemic symptoms (DRESS) was suspected, and pulse therapy with high-dose intravenous methylprednisolone (30 mg/day) was prescribed for 3 days and then 1 mg/kg/day. In addition, the anticonvulsant drug was changed to levetiracetam 50 mg bid.

Her respiratory condition subsequently improved, and she was then transferred back to a regular ward. However, on her 8th day of hospitalization, her eye became icteric, and her direct/total bilirubin increased to 1.8 mg/dL/2.8 mg/dL, before increasing even further to 20.4 mg/dL/31.6 mg/dL. Furthermore, her ALT/AST levels increased to 249 U/L/225 U/L. Meanwhile, an abdominal echo showed mild hepatomegaly. Pulse therapy was then prescribed again, and the patient was also treated with ursodeoxycholic acid and silymarin. She was discharged from the hospital day on the 26th day after her arrival with prescriptions of mycophenolate 2# (180 mg/tab) q12h, tacolimus 1# (1 mg/cap), and prednisolone 2# (5 mg/tab) bid to be taken orally.

## 3. Discussion

DRESS syndrome is an acute, severe, and life-threatening disease with a mortality rate of about 10%. It is more common in adults and only rarely seen in children, in whom it is frequently associated with systemic organ involvement, such as liver dysfunction, renal impairment, and interstitial pneumonitis. Myocarditis, thyroiditis, encephalitis, and type 1 diabetes mellitus have also been reported as manifestations of this syndrome. Other gastrointestinal organs are less frequently affected, but esophagitis, gastritis, enteritis, colitis, and pancreatitis have been reported in recent literature [6].

DRESS usually starts abruptly with maculopapular morbilliform exanthema with a fever of >38 °C as of 2–3 weeks after the introduction of the culprit drug. Pulmonary manifestations are less common and are typically associated with more severe cases [7]. Therefore, when a patient with DRESS initially presents with pulmonary manifestations, a misdiagnosis of pneumonia can occur. *Mycoplasma pneumoniae* infection may induce a DRESS eruption [8], or an upper-airway infection-like prodrome may be detected, suggesting that viral infections may serve as possible triggers for this syndrome [9]. The differential diagnosis between respiratory infections and lung involvement in DRESS is important in these cases. The mycoplasma rapid test in our patient was positive in the ER, resulting in the initial consideration of mycoplasma pneumonia. However, after admission, laboratory tests for mycoplasma IgG and IgM were equivocal and negative, respectively; therefore, mycoplasma infection was able to be excluded. Our patient reported a skin rash 1 week before arrival in the ER, followed by the development of fever and the worsening of the skin rash. We speculate that a viral infection may have triggered the DRESS eruption observed in this patient.

A diagnosis of DRESS can be made based on the diagnostic criteria established by the RegiSCAR group or those established by the Japanese Research Committee on Severe Cutaneous Adverse Reaction, respectively [9,10,11]. Leukocytosis with atypical lymphocytes and eosinophilia of various degrees are unique features of the early phase of DRESS, although leukocytopenia can occasionally precede leukocytosis. Our patient presented with fever and skin rash, and her lab data showed leukocytosis; therefore, mycoplasma pneumonia was suspected initially. However, in tracing back our patient’s past history, it was found that she had a history of epilepsy that been controlled initially under treatment with the anticonvulsant drug sodium valproate, which had subsequently been replaced with lamotrigine 2 weeks after the initiation of which her skin rash first appeared. The patient’s skin rash and drug history were very important clues for diagnosing DRESS.

According to a review article by Shiohara et al. [3], lamotrigine is the fourth most common culprit among anticonvulsant drugs in terms of inducing DRESS. In another study, Newell et al. [12] reported that among 32 children diagnosed with anticonvulsant hypersensitivity syndrome, 12 of them (37.5%) were taking carbamazepine, 11 of them (34.5%) were taking phenytoin, 5 of them (6.25%) were taking phenobarbital, and 5 of them (6.25%) were taking lamotrigine. In still another study, Wang et al. [13] reported that of 57 patients with DRESS induced by lamotrigine, 14 of them (24.6%) were children. This study found a greater predominance of women with lamotrigine-induced DRESS, but in children, we found a greater predominance of lamotrigine-induced DRESS among boys (with a boy-to-girl ratio = 9:7), and we have summarized the characteristics of 16 published cases of pediatric patients with lamotrigine-induced DIHS/DRESS in Table 1. Four of them had DRESS when lamotrigine was given concurrently with sodium valproate.

About 50 to 60% of cases of DRESS with organ involvement occur in the liver, and such DRESS may progress into fulminant hepatitis or hepatomegaly, with hepatic failure being a common cause of death [14].

Systemic corticosteroids have been accepted as the gold standard treatment for ameliorating the clinical symptoms of DRESS. However, they need to be tapered over 6–8 weeks to prevent the relapse of various symptoms [3]. The usage of intravenous immunoglobulin (IVIG) for patients with life-threatening signs such as renal failure or respiratory failure has also been recommended [13,14,15,16]. Meanwhile, some authors have reported the beneficial effects of the concomitant use of N-acetyl cysteine because of its detoxifying capabilities [13]. Alexander et al. reported a dramatic, sustained clinical response to therapeutic plasma exchange after a steroid treatment failed in a pediatric case of DRESS associated with either lamotrigine or bupropion, leading to multiorgan involvement and life-threatening complications of respiratory failure and cardiac arrest [14]. Our patient presented fever and dyspnea initially, and her symptoms progressed to pulmonary insufficiency requiring non-invasive positive pressure ventilator support. Furthermore, our patient developed jaundice with severe liver dysfunction, and the immunosuppressant drugs of mycophenolate and tacolimus were even prescribed after steroid therapy due to hepatic failure. The use of immunosuppressant drugs for DRESS has never previously been reported in the literature. Our patient may thus be the first patient with a case of DRESS treated with immunosuppressant drugs.

The pathogenesis of DRESS remains a matter of speculation, but several theories have been proposed. One theory is that the anticonvulsants are converted into toxic arene oxide metabolites, which are then metabolized by enzymes within the body [17]. Another neoantigen theory speculates that toxic arene oxide reactive metabolites may also alter the cytochrome P450 enzymes, such as those in the liver, skin, kidneys, stomach, intestinal tract, and lungs [18]. In short, it is generally regarded, like other severe drug eruptions, as a T-cell mediated hypersensitivity reaction. Therefore, the clinical resolution of DRESS is associated with a shift away from Tregs to Th 17 cell differentiation [3].

## 4. Conclusions

DRESS should be considered in patients with skin rash and liver function impairment occurring several weeks after the initiation of an anticonvulsant drug. Relatedly, the early recognition and early withdrawal of allergenic drugs is a very important aspect of the management of DRESS. Glucocorticoid therapy is the first-choice treatment, and plasma exchange, IVIG, and immunosuppressant drugs should be considered for multiorgan involvement and life-threatening complications.

## Figures and Tables

**Table 1:** Characteristics of children (<18 year-old) with lamotrigine-induced DIHS/DRESS in published case studies [12,13,14].

Case	Age/Sex	Initial Dose (mg/Day)	Final Dose (mg/Day)	Latency Time (Days)	Concurrent Drugs	Treatment	Outcome
1	11/F	NA	NA	NA	NA	Steroid + IVIG	Cured
2	6/M	NA	NA	10	VPA	No steroid	Cured
3	14/M	NA	NA	52	NA	No steroid	Cured
4	8/M	NA	NA	21	None	Steroid	NA
5	16/F	NA	NA	within 56	NA	NA	NA
6	17/F	50	50	21	None	Steroid	Cured
7	4/F	NA	NA	NA	NA	NA	NA
8	2/F	NA	NA	NA	NA	NA	NA
9	3/M	NA	NA	NA	NA	NA	NA
10	7/M	NA	NA	NA	NA	NA	NA
11	12/M	NA	NA	NA	NA	NA	NA
12	6/M	NA	NA	NA	VPA	No steroid	Cured
13	15/F	50	75	30	VPA 2000 mg/d	NA	NA
14	12/M	25	50	18	VPA	Steroid	Cured
15	4/M	NA	NA	30	NA	IVIG + plasma exchange	Cured
16 *	7/F	50	100	14	NA	Steroid + mycophenolate + tacolimus	Cured

* Our patient. VPA = valproic acid, IVIG = intravenous immunoglobulin, NA = not available.

## Data Availability

The datasets used and/or analyzed during the current study are available from the corresponding author on reasonable request.
